# Multistate Outbreak of Infection with SARS-CoV-2 Omicron Variant after Event in Chicago, Illinois, USA, 2021

**DOI:** 10.3201/eid2806.220411

**Published:** 2022-06

**Authors:** Hillary Spencer, Richard A. Teran, Hannah J. Barbian, Sarah Love, Rachel Berg, Stephanie R. Black, Isaac Ghinai, Janna L. Kerins

**Affiliations:** Centers for Disease Control and Prevention, Atlanta, Georgia, USA (H. Spencer, R.A. Teran);; Chicago Department of Public Health, Chicago, Illinois, USA (H. Spencer, R.A. Teran, S. Love, R. Berg, S.R. Black, I. Ghinai, J.L. Kerins);; Rush University, Chicago (H.J. Barbian)

**Keywords:** COVID-19, 2019 novel coronavirus disease, coronavirus disease, severe acute respiratory syndrome coronavirus 2, SARS-CoV-2, viruses, respiratory infections, zoonoses, Omicron, disease outbreaks, Chicago, Illinois, United States

## Abstract

Bars and restaurants are high-risk settings for SARS-CoV-2 transmission. A multistate outbreak after a bar gathering in Chicago, Illinois, USA, highlights Omicron variant transmissibility, the value of local genomic surveillance and interstate coordination, vaccination value, and the potential for rapid transmission of a novel variant across multiple states after 1 event.

Settings in which adherence to mask wearing and physical distancing is challenging, such as bars and restaurants, pose a risk for transmission of SARS-CoV-2 (*1*). Superspreading events in bars have been linked to subsequent community transmission (*2*,*3*). In early December 2021, the Chicago Department of Public Health was alerted to SARS-CoV-2 infection caused by the Omicron variant (B.1.1.529) in a Louisiana, USA, resident who had attended a party in Chicago, Illinois, USA, over the Thanksgiving weekend (November 2021). The party was held in a large bar that also served walk-in patrons. Many party attendees also attended a dinner at a private event space; the first confirmed Omicron case-patient in Chicago had attended both the bar party and the dinner. We investigated the COVID-19 outbreak after confirmation of infections caused by the Omicron variant in multiple party attendees.

We defined a confirmed SARS-CoV-2 case-patient as a person who tested positive for the virus by molecular or antigen testing of a specimen collected November 25–December 11, 2021, and who had attended the bar event, the dinner, or both on November 27. Probable case-patients were persons experiencing COVID-19-like symptoms (https://ndc.services.cdc.gov/case-definitions/coronavirus-disease-2019-2021) who had attended either gathering but who had no confirmatory testing performed or tested negative. The initial case-patient was interviewed, and additional cases were identified through tracing of household and social gathering contacts; infected persons not associated with the invitation-only events were identified through routine case investigation, in which places of possible transmission are elicited. Contacts were persons who attended either event or both events and had neither confirmed nor probable case status. We verified testing results and vaccination status in reportable disease databases and vaccine registries when possible (vaccination status was self-reported by 2 persons). Available clinical remnant specimens from patients with confirmed cases were submitted to a reference laboratory for whole-genome sequencing. Persons were interviewed by their respective local health departments. We assigned virus lineages by using the PANGO Lineage Assigner (pangolin v3.1.19, pangoLEARN v1/20/22, scorpio v0.3.16; https://cov-lineages.org) and assembled a phylogenetic tree by using PhyML v3.3 (*4*). Our investigation was reviewed by the Centers for Disease Control and Prevention and conducted in accordance with its policies and applicable federal law (45 C.F.R. part 46.102(l)(2), 21 C.F.R. part 56; 42 U.S.C. §241(d); 5 U.S.C. §552a; 44 U.S.C. §3501 et seq).

We identified 15 cases (14 confirmed and 1 probable with negative test results) across 5 states (Figure, panel A); 7 (47%) isolates were sequenced and all were Omicron (B.1.1.529, sublineage BA.1) and closely phylogenetically related (Figure, panel B). Nearly all (14/15, 93%) infected persons had visited the bar, 1 (7%) had attended dinner only, and 1 (7%) had attended both events. Median patient age was 27 (range 23–37) years; 73% were women. Most interviewed persons (86%, 12/14) reported being symptomatic. Of the 11 who reported symptoms and completed the interview, the most common signs/symptoms were cough (82%), fatigue (82%), fever or chills (64%), congestion (64%), and myalgias (55%). None reported loss of smell or taste. In addition, none reported visiting an emergency department and none were hospitalized or died.

**Figure:**
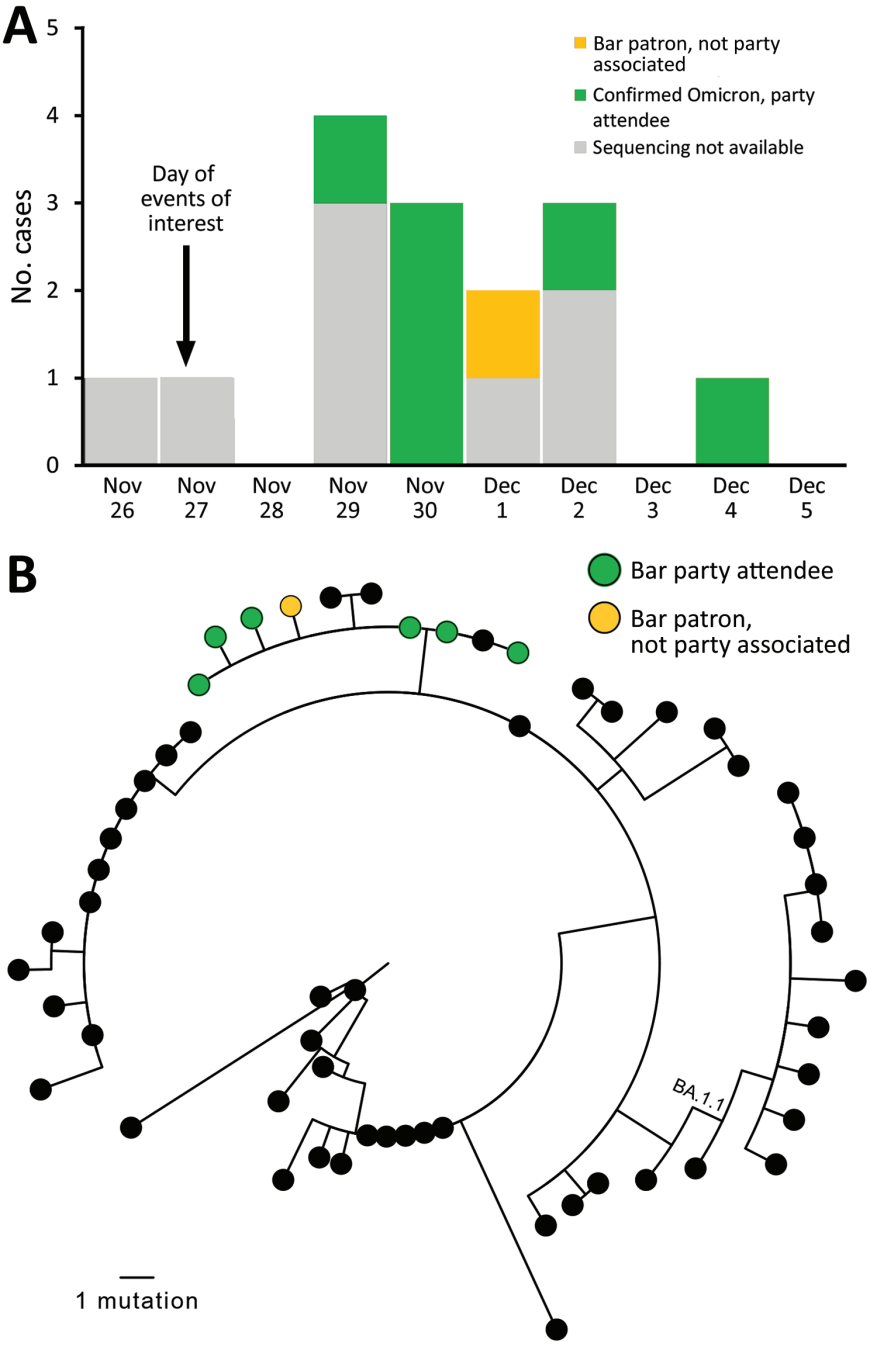
Cases in multistate (Colorado, Illinois, Louisiana, Missouri, Michigan) outbreak of infection with severe acute respiratory syndrome 2 Omicron variant after event in Chicago, Illinois, USA, November–December 2021. A) Cases over time. Sequencing results are shown, if available. B) Genetic relatedness of viruses isolated. Maximum-likelihood phylogeny of 7 sequenced Omicron samples in bar-associated outbreak (green and yellow) with 50 contextual sequences (black). Contextual sequences are a random sample of Omicron BA.1 and BA.1.1 sequences selected from all Omicron sequences in GISAID (https://www.gisaid.org) that were collected in the United States or before December 11, 2021, and had >90% genome coverage. Random selection was performed by using CLC Genomics Workbench (QIAGEN, https://www.qiagen.com). No contextual sequences were from Illinois. GISAID accession numbers for all included sequences are listed in the Appendix. One outbreak-associated specimen was sequenced by a private laboratory and not uploaded to GISAID. Full-genome sequences were used for PhyML phylogenetic analysis (*4*), excluding 250 bp from genome ends and an error-prone region (reference positions 21492–21935). Outbreak sequences were identical to each other or contained a single-nucleotide substitution (T12000C, T22813G, T25414C) and clustered (with 3 contextual sequences) in a clade diverged by 2 nts from the closest other sequences. The 2 nt substitutions that defined the outbreak branch (C11950T, C28472T) were present in just 5.2% of contemporaneous Omicron sequences from the United States available on GISAID, indicating that all available outbreak sequences were closely genetically related.

Most (80%, 12/15) persons were fully vaccinated (i.e., >14 days after receiving 2 doses of the Pfizer-BioNTech [https://www.pfizer.com] or Moderna mRNA vaccine [https://www.moderna.com] or 1 dose of the Johnson & Johnson/Janssen vaccine [https://www.jnj.com]). Of those, 25% (3/12) had received a booster 13–32 days before the event). Median time since receipt of most recent vaccine among 9 persons fully vaccinated but not boosted was 258 (interquartile range 209–279) days. Two infected persons had prior SARS-CoV-2 infection (113 and 468 days since previous infection); both were fully vaccinated (1 before and 1 after infection). Three (20%) infected persons were either unvaccinated or partially vaccinated. Five infected persons visited the bar as members of the public (not party attendees); of those 5, Omicron was confirmed in the 1 specimen that was available for sequencing. Identifying this variant at a time when it was not widely circulating prompted interstate notification for this out-of-state person.

This outbreak involving transmission in a bar between close social contacts and non–party-associated bar patrons demonstrates the high potential for Omicron transmission in indoor settings for which consistent mask use and distancing are challenging. Although no persons in this outbreak experienced severe disease, most were young and fully vaccinated. Local capacity for genomic sequencing, conducted across 7 laboratories in 5 states (Colorado, Illinois, Louisiana, Missouri, Michigan), enabled identification of linked case-patients beyond invited attendees who may have been excluded from traditional epidemiologic investigations. 

Outbreak investigation limitations include incomplete identification of, or nonresponse from, dinner and party attendees; limited availability of clinical remnant specimens; and inability to estimate attack rates among persons in the bar. This outbreak highlights Omicron transmissibility; the value of local genomic surveillance capacity and interstate coordination; the value of vaccination for reducing the likelihood of severe disease; and the potential for rapid, widespread transmission of a novel variant across multiple states from 1 event over a holiday weekend.

Appendix GISAID accession numbers for all sequences included as part of investigation of multistate outbreak of infection with SARS-CoV-2 Omicron variant after event in Chicago, Illinois, USA, November–December 2021.
